# An Innovative Assessment of the Dynamics of Facial Movements in Surgically Managed Unilateral Cleft Lip and Palate Using 4D Imaging

**DOI:** 10.1177/1055665620924871

**Published:** 2020-05-18

**Authors:** Shyam Gattani, Xiangyang Ju, Toby Gillgrass, Aileen Bell, Ashraf Ayoub

**Affiliations:** 1 Glasgow Dental School, School of Medicine, College of Medical, Veterinary and Life Sciences, University of Glasgow, Glasgow, United Kingdom; 2 Medical Devices Unit, Department of Clinical Physics and Bioengineering, National Health Service of Greater Glasgow and Clyde, United Kingdom; 3 Glasgow Dental Hospital & School, Glasgow, United Kingdom; 4 Oral Surgery, Glasgow University Dental Hospital & School, Glasgow, United Kingdom; 5 Scottish Craniofacial Research Group, Glasgow University Dental Hospital & School, School of Medicine, College of Medical, Veterinary and Life Sciences, University of Glasgow, Glasgow, United Kingdom

**Keywords:** 4D imaging, cleft lip and palate, facial asymmetry, facial motion, smile

## Abstract

**Objective:**

Assess facial asymmetry during maximum smile in patients with surgically managed unilateral cleft lip and palate (UCLP), using a dynamic 3-dimensional (3D) imaging (4-dimensional) system.

**Design:**

Prospective 2 cohort comparative study.

**Methods:**

Twenty-five surgically managed UCLP cases and 75 controls at 8 to 10 years of age were recruited. Facial movements during maximum smile were recorded using video stereophotogrammetry at a rate of 60 3D facial images per second. Maximum smile took approximately 3 seconds and generated 180 3D facial images for the analysis. A generic facial mesh which consists of more than 7000 quasi landmarks was used for the assessment of facial asymmetry at 5 key 3D frames representing the pattern of maximum smile.

**Results:**

Statistically significant differences were seen regarding the magnitude of facial asymmetry between the UCLP group and the noncleft controls. Higher average asymmetry in the UCLP group was seen in the 3D frame midway between maximum smile and rest (frame 4) followed by the frame at peak expression of maximum smile (frame 3). The average magnitude of nasolabial asymmetry of the control group was within 0.5 mm in comparison with the UCLP cases which was about 1.8 mm.

**Conclusion:**

This study provided for the first time, an objective tool for analysis of the dynamics of muscle movements which provided an unprecedented insight into the anatomical basis of the residual dysmorphology. The research demonstrates the limitations of the primary lip repair in achieving symmetrical results and underpins the required refinements to improve the quality of surgical repair of cleft lip.

## Introduction

Cleft Lip and palate (CLP) is a craniofacial anomaly that affects 1 in 700 children per year (Thomason & Dixon, 2009). The focus of the surgical repair of cleft lip is to improve lip functions and facial aesthetics. Despite surgical correction, facial asymmetry is not fully eliminated (Seaward et al., 2015). The residual asymmetry results from the formed scar tissue, muscular pull, and relatively thinner tissues at the surgical site (Otero et al., 2012). In addition to the static facial asymmetry (in a still facial image), the distorted facial movements after the surgical repair of cleft lip have a profound psychosocial impact (Shaw, 1981). In this era of a high-pressured celebrity culture, where appearance is considered as a gateway to social acceptance, even minor asymmetries on the face are associated with negative social responses such as unwarranted staring and isolation at school and among peers (Bradbury, 2012). This ultimately leads to a sense of shame, anxiety, depression, and more importantly a lack of ego development in the affected children (Bradbury *&* Hewison, 1994).

Previous studies have shown that subjective interpretations of surgical outcomes and the need for further lip surgery are unreliable and inaccurate. It has been seen that surgeons’ agreement among themselves regarding the severity of the nasolabial deformity (Asher-McDade et al., 1991) or the outcomes of surgery (Trotman et al., 2007; Trotman et al., 2011) were low.

The objective evaluation of facial movements in the surgically managed patients with CLP included studies on photographs and video recordings. These techniques only provided a 2D analysis and did not accurately quantify the complexity of 3-dimensional (3D) dynamic facial expressions. Gross et al. (1996), in a study comparing 2D and 3D methods of facial movement, inferred that measurements in 2D underestimated analogous 3D measurements by 43%. Several studies have been conducted using 3D images of static faces at rest and at maximum facial expressions using laser scanning (Schwenzer-Zimmerer et al., 2008; Djordevic et al., 2014) and stereophotogrammetry (Trotman et al, 2007; Meyer-Marcotty et al., 2010; Bell et al., 2014; Patel et al., 2015). Human faces however are rarely static in day to day activities. The assessment of the dynamics of facial movements requires that facial expressions to be recorded in real time 4-dimensional (4D) to assess morphological characteristics at various time intervals. Static 3D capture of facial movements does not record these characteristics and therefore does not allow the analysis of the dynamics of facial movements during facial expressions.

There is a paucity of 4D research with regard to the nasolabial movements on CLP cases. Hallac et al. (2016) assessed facial asymmetry in surgically managed cleft lip and/or palate patients using 4D video stereophotogrammetry. However, the assessment was confined to only 13 facial landmarks. The asymmetry of facial movements was assessed by measuring the displacement of these specific landmarks. The differences in the speed of displacement of these landmarks between the cleft and noncleft sides were measured. The motion path of landmarks was compared between cleft and noncleft sides to provide a geometric analysis of asymmetry. However, the landmark-based analysis is limited in describing the morphological changes of the facial surfaces at certain points and the rich data captured in 3D was not fully utilized in their study.

Trotman et al. (2005, 2011, 2013) studied the dynamics of facial expressions through tracking a set of markers which were directly placed on the patients’ face. Changes in inter-landmark distances in cleft patients were compared to that in noncleft participants. The changes in inter-landmark distances were used to study facial movements pre and postprimary surgical repair of the cleft lip. The application of the skin markers warrants the need for a high level of patient cooperation and operator skill for the accurate positioning of the anatomical points during various imaging sessions. Once more, landmark-based analysis has its limitations as explained above.

The objective of this study was to assess the magnitude of facial asymmetry during maximum smile in surgically managed unilateral cleft lip and palate (UCLP) cases. A novel method was introduced to measure the magnitude of whole facial and regional asymmetries in patients with UCLP during the maximum smile (ie, from repose to peak expression) in comparison to noncleft controls.

The hypothesis tested in this study:

- There is no statistically significant difference in magnitude of facial asymmetry during maximum smile between the UCLP group and control group.

## Material and Methods

Ethical approvals were obtained (REC reference: 16/NE/0246) and the NHS R&D (R&D Reference GN16OD291). All procedures including filing and storage of data were adhered to according to the guidelines and policies set forth by health authorities.

### Sample Size Calculation

Based on information obtained from previous studies by Bughaigis et al. 2014 and Kuijpers et al. 2015, a standard deviation of 1 and an effect size of 0.7 for average asymmetry scores was observed. It was calculated that 22 participants in the UCLP group and assuming a ratio of 3:1 for controls: cases (Hennessy et al., 1999), 66 noncleft volunteers would need to be recruited for the above study assuming a power of 80%, in order to detect differences between the UCLP group and the noncleft control group.

**Figure 1.**
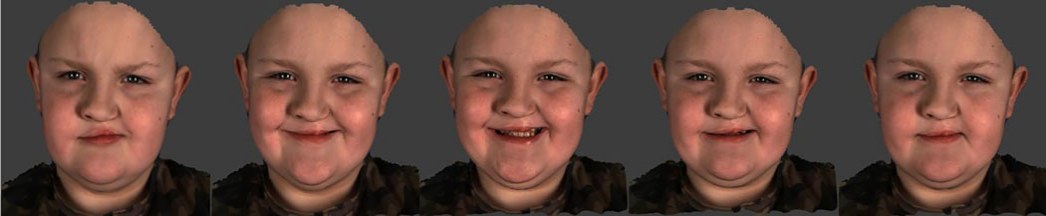
Three-dimensional image sequence of the maximum smile expression in a cleft lip patient.

Therefore, the study sample consisted of 25 patients with UCLP, aged 8 to 10 years, 75 age, and sex-matched noncleft volunteers recruited as controls. All the patients had complete UCLP, were managed by the same clinical team, and followed a standard surgical protocol; Millard lip repair was carried out between 3 and 6 months of age. Each participant was imaged using a 4D video stereophotogrammetry device, the Di4D capture system (Dimensional Imaging). The system consisted of 2 gray scale cameras (Model aVA 1600-65 km/kc; resolution 1600 × 1200 pixels; sensor model KAI-02050; Kodak) and 1 color camera that captures images at a rate of 60 image frames/second using a light source (Model DIV401-DIVALITE; Kino Flo Corporation). The Di4D imaging system captures the maximum smile expression at a rate of 60 frames/second. The maximum smile takes about 3 seconds which generates 180 3D facial images (Figure 1). The imaging system is based on passive stereophotogrammetry which allows automatic tracking of facial landmarks throughout the sequence of facial expression frames. The clinical validity of the automatic tracking of facial landmarks has been studied by Al-Anezi et al. (2013) and applied clinically (Shujaat et al., 2014 and Al-Hiyali et al., 2015).

The maximum smile was chosen in this study due to its high reproducibility (Johnston et al., 2003). Prior to each imaging session, participants were shown how to perform the maximum smile by means of visual cue cards and observation-based training. Each participant was then asked to sit in a relaxed and upright position, 95 cm away from the camera in front of a blue screen before imaging was undertaken. Participants were asked to start the expression at a state of rest, keeping their lips in light contact and ensuring contact of posterior teeth and slowly progressing to a maximum smile by showing their teeth and stretching the corners of their lips as much as possible before coming back to a state of rest. If participants moved during the imaging session, the procedure was repeated.

### Image Processing

For the analysis of asymmetry of maximum smile, a generic face mesh was applied. A generic mesh is a universally applicable facial surface representing morphological information of an average face, which consists of common morphological characteristics within the population. The generic mesh consists of more than 7000 triangulated vertex “points,” whose 3D coordinates are fixed.

**Figure 2.**
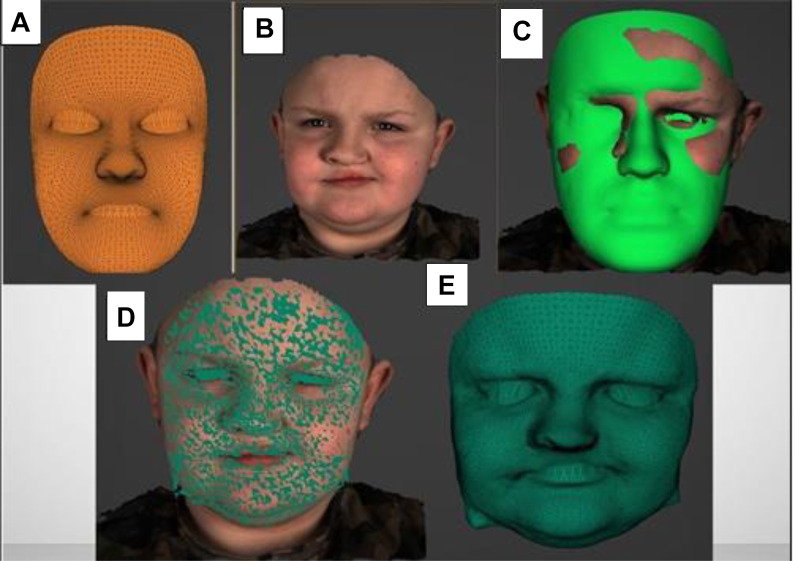
Conformation process. A, Wireframe model of generic mesh; (B) Three-dimensional frame of initial resting face; (C) Conformation process; (D) Conformed mesh over 3D frame; (E) Conformed mesh surface model. 3D indicates three dimensional.

This generic mesh was elastically deformed in a process known as mesh conformation to represent the patients/participants underlying facial morphology, thus creating a conformed mesh specific to each patient. This process of conformation was started by manually identifying and digitizing 29 facial landmarks (Table 1), Al-Hiyali et al. (2015) on the generic mesh as well as on the 3D facial model on the resting frame at the beginning of the maximum smile. The landmarks were then automatically tracked throughout the entire sequence of 3D facial images. The landmarks initiated the 3D mapping of the mesh on the face which was followed by computerized elastic deformations to “wrap” the mesh around the anatomical morphology of the face (Figure 2). We validated the accuracy of this process (Al Mukhtar et al., 2017). The conformed face mesh was tracked along the subsequent frames of the maximum smile expression. The accuracy of the tracking was validated (Al Anezie et al., 2015). Analysis of the errors of landmarking (intraoperator reliability) was carried out by repeating, a week apart, the digitization of the facial landmarks on 10 randomly selected cases, by the same operator. The difference in landmarking was statistically analyzed using Student *t* test.

**Table 1.** Names and Definitions of Landmarks Manually Digitized on the First 3D Frame.

Landmark number	Landmark name	Description
1 and 2	Superciliary points	Points located above the most superior part of the eyebrows
3 and 6	Exocanthion	Points at outer corner of the eye fissure
4 and 5	Endocanthion	Points at the inner corner of the eye fissure
7 and 8	Zygion	Most prominent point on the cheek area beneath the outer canthus and slightly medial to the vertical line passing through it.
9	Nasion	The midpoint on the soft tissue contour of the base of the nasal root at the level of the frontonasal suture
10	Pronasale	The most anterior midpoint of the nasal tip
11	Pogonion	The most anterior midpoint of the chin
12	Subnasale	Midpoint of angle at the columella base where the lower border of the nasal septum and the surface of the upper lip meet
13 and 14	Subalare	Point on the margin of the base of the nose where the ala disappears into the upper lip skin
15 and 16	Alar curvature	Most lateral point on the curved base line of each ala
Landmark number	Landmark name	Description
17 and 18	Cheilion	Point located at the corner of each labial commissure
19	Labrale superius	The midpoint of the vermilion line of the upper lip
20	Labrale inferius	The midpoint on the vermilion line of the lower lip
21 and 22	Crista philtri left and right	Peak of cupids bow left and right
23 and 24	Corresponding lower lip points to crista philtri left and right	Corresponding points on lower lip to crista philtri left and right
25 and 26	Upper middle lateral lip points	Midpoints located between cheilion and crista philtri left and right
27 and 28	Lower middle lateral lip points	Midpoint between cheilion and 23 and between cheilion and 24
29	Glabella	Midline point between eyebrows

Abbreviation: 3D, 3-dimensional.

Of the 180 generated 3D facial frames of each facial expression (maximum smile), 5 frames were selected by the operator for the assessment of asymmetry of maximum smile. The 5 frames were as follows:

Frame 1–Initial resting face (start of the maximum smile expression)

Frame 2—Frame between rest and peak expression (arithmetic midpoint between frame 1 and frame 3)

Frame 3—Peak/maximum expression (selected based on the frame that showed maximum stretching of the lip commissures)

Frame 4—Frame between peak/maximum expression and final resting position (arithmetic midpoint between frame 3 and frame 5)

Frame 5—Final resting face (end of the maximum smile expression)

### Assessment of Facial Asymmetry

For each of the 5 frames (3D images) obtained per patient/control, a mirror image was created by reflecting the 3D conformed mesh on a reference plane (an arbitrary mathematical plane acting as a mirror). Therefore, images with right-sided cleft become left sided and vice versa. This procedure of mirroring was carried out for each the 5 selected frames of the captured 3D sequences of facial images during maximum smile for both the UCLP cases and the control group. To standardize the analysis, the images of the right-side clefts were reflected so that the cleft was on the left side for all the cases.

Each of the 5 selected 3D facial frames and their mirror images were superimposed using Partial Ordinary Procrustes Analysis. This algorithm produced optimum superimposition through aligning the images using components of translation and rotation.

The asymmetry scores were ascertained by measuring the distances between corresponding vertices of the 3D surface meshes of the superimposed images. In perfect symmetry, the distance between the original and mirrored image equaled zero. The method provides an accurate evaluation of asymmetry which has been validated by our team (Al Rudainy et al., 2018a, 2018b, 2019). This resulted in 5 asymmetry scores (1 for each of the 5 frames) per case. The statistical significance of the measured asymmetries of each of the 5 selected frames was evaluated using was analysis of variance test. These 5 asymmetry scores were averaged across each group resulting in an average asymmetry score for cleft cases and an average asymmetry score for controls.

A major disadvantage of using total face asymmetry scores is that the larger asymmetry of certain anatomical regions of the face may be diluted by the smaller asymmetry scores of other parts of the face. In order to overcome this limitation, regional asymmetry scores specific to the nasolabial region were measured.

The asymmetry scores were displayed in colors ranging from dark blue to red. The color-coded map represented the size of the distances between the corresponding vertices of the superimposed meshes. As the distance between corresponding vertices has increased, the color changed from blue to sky blue to yellow to orange and finally red. The red color indicated a higher area of asymmetry. Directionality of asymmetry was also assessed in the x, y, and z directions or the horizontal, vertical, and anteroposterior directions, respectively.

## Results

### Errors of the Method

No statistically significant differences were detected between the repeated digitization of the anatomical landmarks (*P* < .05).

### Magnitude of Facial Asymmetry

In the UCLP group, the male to female ratio was 1.2:1 (14 males:11 females), and the mean age was 8.68 years with a median age of 9 years; in the control group, the male to female ratio was 1.1:1 (38 males: 37 females), and the mean age was 8.84 years with a median age of 9 years.

**Figure 3.**
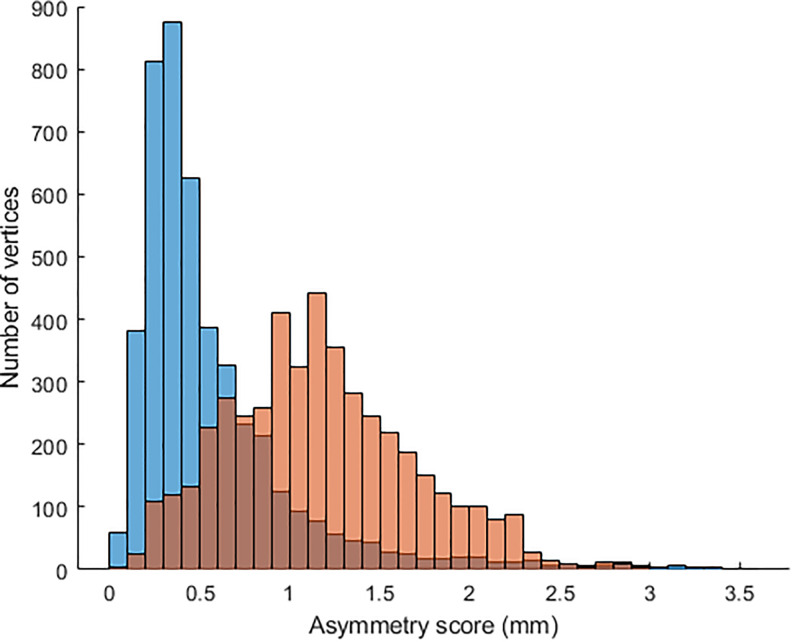
The average magnitude and the distribution pattern of nasolabial asymmetry of the control group which was within 0.5 mm in comparison with the UCLP cases which was about 1.8 mm. UCLP indicates unilateral cleft lip and palate.

Figure 3 is a graph comparing the average asymmetry scores of each of the 5 frames in the surgically managed UCLP cases and control group. The average asymmetry scores were seen to be higher in the surgically managed UCLP cases than the controls (*P* < .05). The differences in the asymmetry scores in all frames between the 2 groups were significant (*P* < .001). The trend of the asymmetry scores of the control group differed from that of the surgically managed UCLP cases. In the control group, frame 3 illustrates the average magnitude and the distribution pattern of nasolabial asymmetry of the control group which was within 0.5 mm in comparison with the UCLP cases which was about 1.8 mm.

In the UCLP group, the maximum asymmetry was detected in frame 4, midway between maximum smile and final resting pose. Residual asymmetry was measured at the final resting frame which “frame 5” was significantly different from the rest 3D frames of the maximum smile.

In UCLP cases, the following were the correlations between the frame of the 3D facial image and at rest and the other frames, respectively; frame 2:0.80; frame 3:0.68; frame 4:0.82; frame 5:0.86. These were statistically significant (*P* < .0001). The same pattern of strong significant correlations was detected between the rest frame and the others.

The null hypothesis was rejected, indicating that there was a statistically significant difference in the magnitude of facial asymmetry between the surgically managed UCLP cases and the controls. The asymmetry scores of the lower lip at the third and fourth frames were significantly higher in the UCLP cases than that of the controls.

**Figure 4.**
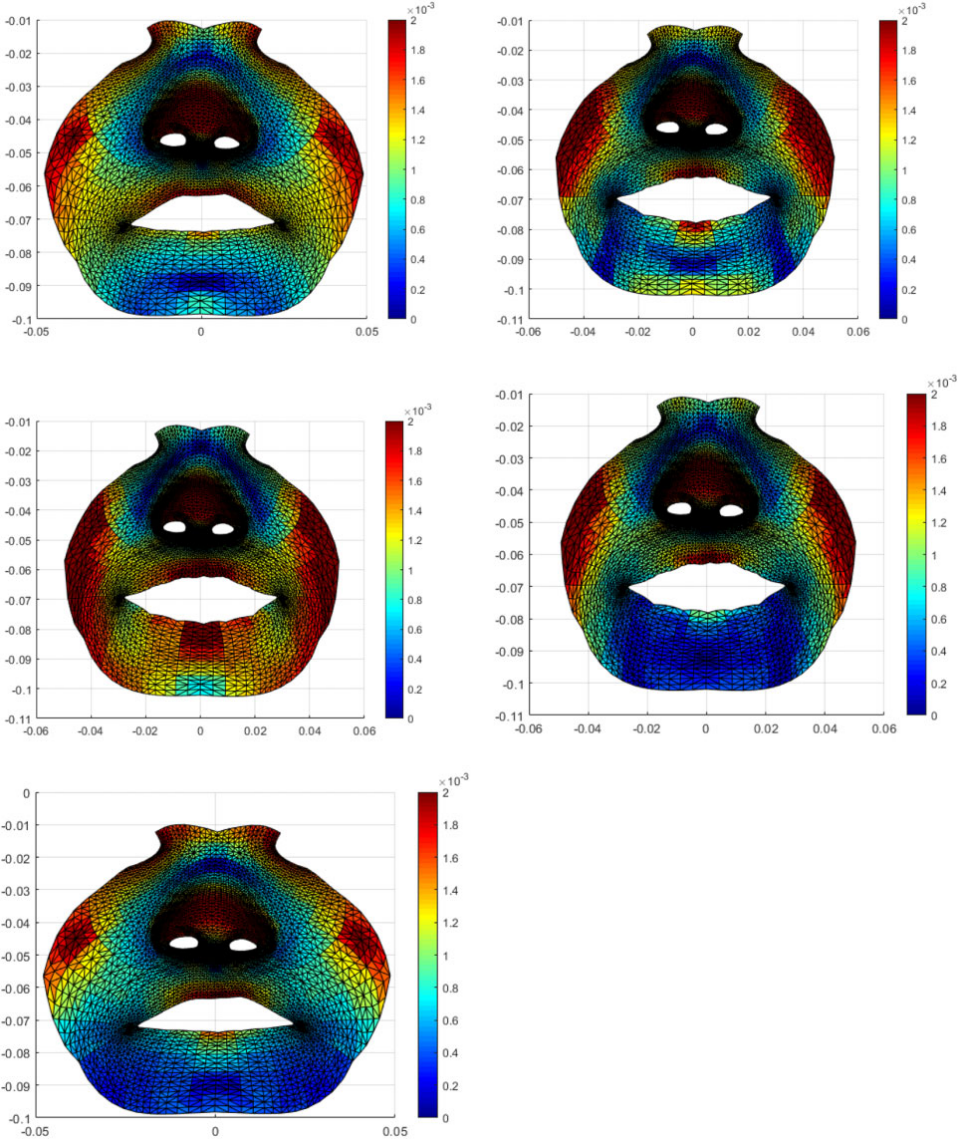
Colour maps showing the average asymmetry of the maximum smile in UCLP patients of the five selected frames. Red areas represent maximum asymmetry. Frame 1 shows mild asymmetry of the vermilion border of the upper lip at rest. Frame 2 shows a more intense red colure affecting a wider region of both the upper and lower lips. Frame 3—The peak expression shows an increased asymmetry which became more obvious on the lower vermilion border. Frame 4—Residual asymmetry mainly of the upper vermilion. Frame 5 shows minimal asymmetry similar to the first frame. UCLP indicates unilateral cleft lip and palate.

In UCLP cases, the nasolabial asymmetry was detected in the mediolateral “x plane,” vertical “y plane,” and in anteroposterior direction “z plane” in each of the 5 frames during maximum. It was noticed that asymmetry was accentuated during performance of the facial expression and frame 3 and 4 demonstrated higher asymmetry especially in areas of the vermillion border of the upper and lower lip and the alar regions. The magnitude of asymmetry at these frames was statistically significant in comparison to the first frame recorded at rest (*P* < .05). This confirms that the maximum smile exaggerates the asymmetry which is noted at the rest pose (Figure 4).

**Figure 5.**
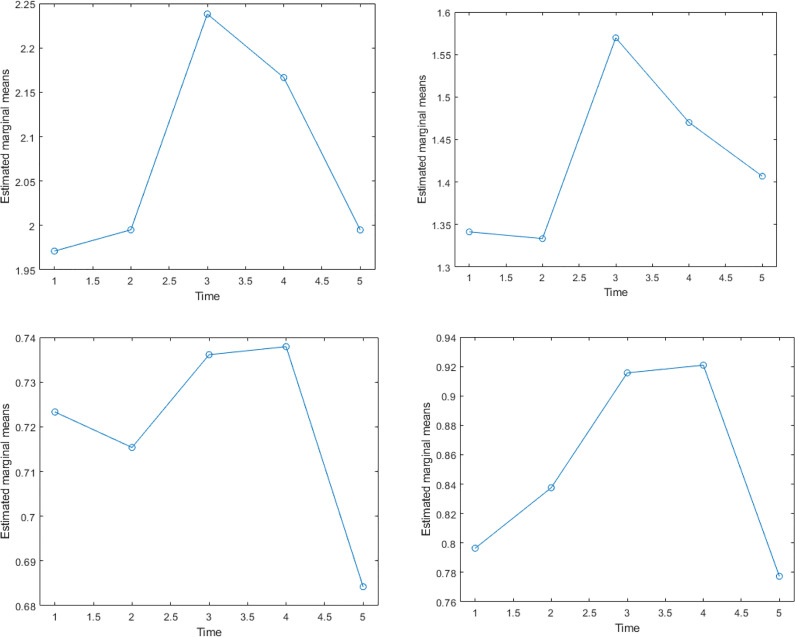
Diagrammatic representation of the dynamics of facial asymmetry throughout the 5 frames with maximum smile of the UCLP cases. Top left shows the total asymmetry, top right shows the asymmetry in the X direction, the bottom left, and right show the asymmetry in the Y and Z directions accordingly. UCLP indicates unilateral cleft lip and palate.

The asymmetry of maximum smile became more apparent when the directionality of the lip movement was considered in the analysis (Figure 5). Mediolaterally, the deviation toward the cleft side was noticed and increased especially in frame 3 and frame 4 of the maximum smile, particularly in areas around the vermillion borders of the upper and lower lips. Vertical upward asymmetry was noticed in frames 3 and 4 (as compared to the other frames) in areas around the vermillion border of the upper and lower lips, the alar regions, and around the nostrils. There was a clear tendency for more asymmetry at the fourth frame as the face starts to relax after maximum smile. The increased asymmetry in the anteroposterior direction (Z plane) was noticed in areas around the vermillion borders of the upper and lower lips, the alar regions, the philtrum, and columella and around the nostrils. Likewise, the fourth fame showed more asymmetry.

It was clear that the residual asymmetry in all 3 spatial planes was seen to gradually increase as the patients performed the maximum smile and decreased toward the final resting state. Visualizing asymmetry using the color maps aided in assessing the directionality of asymmetry as well as understanding the mechanism of asymmetry of maximum smile, frame by frame in each anatomical area within the nasolabial region of the UCLP cases.

## Discussion

Asymmetry is significantly pronounced in unilateral craniofacial deformities. This study provided for the first time, an objective, dynamic analysis of the asymmetries of facial expression in patients with UCLP. For the accurate assessment of facial asymmetry, the innovative application of a conformed generic facial mesh on 4D images was considered. The use of the entire facial surface for assessing facial movements and evaluating the asymmetry is an innovation in this study. Previous studies in 3D (Trotman et al., 2007) and 4D (Hallac et al., 2017) analyzed asymmetry or facial movement using a specific number of anatomical landmarks on the face which neither described fully the pattern of facial movements nor did they disclose the regional asymmetry of the morphological areas of the face between these individual landmarks. On the other hand, the conformed mesh enabled the analysis of the entire surface of the face and helped in establishing regional asymmetry and the directionality of asymmetry in the 3 spatial planes.

Very few 4D studies have been conducted previously on patients with cleft lip (Trotman et al., 2005; Trotman et al., 2011; Trotman et al., 2013; Hallac et al., 2016). None of these studies have evaluated the asymmetry throughout the course of facial expression. This is the first time to record the asymmetry of the dynamics of maximum smile which provided an unprecedented insight into the anatomical basis of the recorded residual dysmorphology. The rational of assessing facial movement at key frames during maximum smile is the fact that the asymmetry of facial morphology at each of the 5 frames have a specific group of muscles responsible for the movement of the nasolabial region. The assessment of the functional symmetry at these frames provides an insight on the mechanism of action and dysfunction of the related group of muscles. This would inform and guide cleft repair and the surgical correction of the residual morphologic and functional deformities.

Performance of the maximum smile involves the activity of the perioral muscle group, starting with the contraction of the levator muscles which assist in the movement of the upper lip toward the nasolabial fold followed by the contraction of the risorius, zygomaticus major and minor, and the buccinator which helps the lips to move further superiorly and laterally (Manjula et al., 2015). The question arises as to why the asymmetry is most pronounced in the frame midway between peak expression and final resting position (frame 4) which was detected in the UCLP cases. The first 2 muscles participating in the maximum smile are the levator labii superioris (LLS) and the levator labii superioris alaeque nasi (LLSAN). The LLS muscle consists of 3 heads each showing different origins and insertions. The angular head consists of the medial fibers originating from the upper part of the frontal process of the maxilla below the infraorbital foramen. These divide into 2 slips of muscles, the first attaching to the greater alar cartilage and ala of the nose and the second in to the muscles of the upper lip, blending with the orbicularis oris. The intermediate or infraorbital head of the LLS consists of muscle fibers that originate below the orbit immediately above the infraorbital foramen and attach to the muscular portion of the upper lip between the levator anguli oris and the angular head muscle fibers. The zygomatic head originates in the malar process of the zygomatic bone and inserts in to the modiolus near the corner of the mouth. The deep layer of the LLSAN muscle is located laterally to the transverse nasalis muscle (Hur et al., 2010). The restoration of the normal anatomy and the intricate relationships of the LLS and the LLSAN with the facial alar crease, the nasal vestibule, and the orbicularis oris is essential during primary repair of a cleft lip. Therefore, it is not unreasonable to conclude that the incomplete mobilization, rotation, and the approximation of these group of muscles would contribute to asymmetry during maximum smile.

Additionally, scarring within and around the 2 muscles would compromise the range and speed of muscle movements and may contribute to the measured facial asymmetry in frame 3 or the peak expression in the UCLP group. But assessment of the velocity of the movement of upper lip muscles movement was beyond the scope of this study. Maximum facial asymmetry was also observed in frame 3 in the noncleft controls. Asymmetry at frame 3 (peak expression) of the maximum smile in the UCLP and the control group indicates that facial expression accentuates facial asymmetry.

The peak smile frame also involves the contraction of the zygomaticus major, the zygomaticus minor, and the risorius muscles which help to raise the corner of the mouth producing the stretching of the lips during maximum smile. As these groups of muscles are uninvolved during the cleft lip repair surgery, asymmetry in frame 3 was seen to be comparatively less than in frame 4 in the UCLP cases.

In frame 4, following the peak expression of smile, high asymmetry scores (in the UCLP group) could be attributed to the fact that the poorly approximated levator muscles are working to bring the upper lip back to its original resting position. The high asymmetry scores is attributed to the “residual force enhancement theory” which states that the force of skeletal muscles immediately following eccentric or lengthening or stretching movement is higher than the force produced during isometric contraction (Campbell & Campbell, 2011). This seems to suggest that following stretching of these muscles, instead of transitioning into a state of relaxation; muscles hold high tension/force prior to the final resting stage. Muscles may thus be working in an increased energy expenditure environment occurring at the cost of reduced blood flow. The asymmetry of the lower lip of the UCLP is due to the distorted muscle dynamics secondary to the asymmetry of the upper lip that was measured at rest and during movement.

In summary, the primary objective of the primary cleft repair is to ensure optimum reconstruction of these muscles both in terms of functionality and morphology. Surgically managed UCLP cases had a more restricted upper lip movement as compared to noncleft volunteers. This asymmetry of upper lip movement contributes to the limited lateral movements around the upper lip. The adequate mobilization of the disrupted nasolabial muscles in UCLP is essential and perhaps the microscopic repair of these muscular bundles may be considered to improve lip function and to reduce the asymmetry of facial expressions.

## Conclusion

This study provided, for the first time, a useful instrument for the analysis of the dynamics of facial muscle movements which disclosed the mechanism of the asymmetry of facial expression during maximum smile. This is the first study documenting the asymmetry of the dynamics of maximum smile throughout the course of this facial expression which provides an insight into the anatomical basis of the residual asymmetry following cleft lip repair. This should inform the primary repair of cleft lip and guide the surgical techniques for the secondary surgery to deal with the residual nasolabial dysmorphology and dysfunction. The study presents an objective tool to evaluate and compare the outcomes following cleft lip repair, with a specific focus on the dynamics of the nasolabial muscle function. This could be used for comparative analysis of various surgical techniques and for outcome measures of various cleft centers.
